# Analysis of the benefit of gonadotropin-releasing hormone agonist treatment in premenopausal women undergoing hematopoietic cell transplantation

**DOI:** 10.1038/s41598-023-40778-2

**Published:** 2023-09-04

**Authors:** Ruxue Han, Ziyi Song, Huiling Li, Chaohua Wang, Leping Zhang, Xin Yang

**Affiliations:** 1 Department of Gynecology and Obstetrics, Peking University People’s Hospital, Beijing, China; 2 Department of Pediatrics, Peking University People’s Hospital, Beijing, China

**Keywords:** Endocrine system and metabolic diseases, Haematological cancer

## Abstract

Gonadotropin-releasing hormone agonist (GnRHa) appears to exhibit ovarian protection during chemotherapy for malignant tumors. The purpose of this study was to analyze the benefits of GnRHa in premenopausal women undergoing hematopoietic cell transplantation (HSCT). Candidates for myeloablative chemotherapy HSCT requiring fertility preservation in the Gynecological Endocrinology Clinic of Peking University People’s Hospital from December 2011 to December 2021 were retrospectively analyzed. Patients who chose to receive GnRHa treatment were given at least 2 courses of a 3.75-mg dose of a GnRHa before myeloablative chemotherapy, and patients who chose not to receive GnRHa treatment were included in the control group. All patients were monitored for menstruation return and menopause-related symptoms, and ovarian function tests [follicle-stimulating hormone (FSH), luteinizing hormone, and estradiol] were performed 6–12 months after HSCT. In addition, we assessed the vaginal bleeding of patients in the laminar air-flow room (LAFR). A total of 234 cases were included in this study: 77 cases in the treatment group and 157 cases in the control group. The incidence of vaginal bleeding in the LAFR in the treatment group was significantly lower than that in the control group (24.68% vs. 79.62%, *P* < 0.001). The menopausal symptoms of the patients in the treatment group were reduced after transplantation (46.75% vs. 19.75%, *P* < 0.001). There was no difference in visible follicles by follow-up ultrasound in the two groups after HSCT (16.88% vs. 13.38%, *P* = 0.474). The level of FSH at 6–12 months after transplantation was lower (98.00 mIU/ml vs. 117.53 mIU/ml, *P* = 0.001). The proportion of patients with FSH < 40 mIU/ml did not differ between the two groups. One patient in the treatment group recovered spontaneous menstruation, while none recovered spontaneous menstruation in the control group (1.30% vs. 0%, *P* = 0.329). The use of GnRHa may relieve menopause-related symptoms and reduce vaginal bleeding in the LAFR and breakthrough bleeding after transplantation. GnRHa treatment can reduce the level of FSH after myeloablative chemotherapy, but it cannot reduce the incidence of premature ovarian failure in women of reproductive age following myeloablative HSCT.

## Introduction

Hematopoietic stem cell transplantation (HSCT) is a well-established treatment for many congenital or acquired diseases of the hematopoietic system and several other life-threatening conditions^1^ that can effectively improve the survival rate of patients. With the advancement of medical technology, the long-term survival rate of such patients has increased significantly^2^. An increasing number of people are pursuing quality of life on the basis of surviving, and have the need to establish a family or fertility needs. Huang and colleagues at Peking University established the “Beijing Protocol” for haplo-SCT using myeloablative conditioning (MAC) regimen^3^, solving the problem of insufficient hematopoietic stem cell donors. Patients with nonhematological malignancies (such as severe aplastic anemia) or patients who are older than 55 years or exhibit impaired vital organ function can choose the reduced intensity conditioning (RIC) regimen. However, the commonly used MAC regimens and RIC regimen both include high-dose chemotherapy and total-body irradiation. The chemotherapy dosages used in these combinations are very gonadotoxic. It has been reported that the prevalence rate of ovarian insufficiency exceeds 90–100% of female patients who have received HSCT following MAC^4–6^. Our team's single-center preliminary study found that the incidence of premature ovarian failure (POF) after HSCT in female patients over 20 years old can reach 100%^7^. Studies have shown that HSCT is an independent risk factor for POF^8^.

Although impaired ovarian function after HSCT is a clinically recognized phenomenon, there are few studies on the protection of ovarian function, with most of these investigations focusing on the cryopreservation of oocytes, embryos, and ovarian tissues^9, 10^. These studies partially address fertility issues associated with ovarian failure but do not apply to patients who must undergo transplants in the short term or who are physically or financially disadvantaged.

Due to iatrogenic POF, patients also face the early onset of menopausal symptoms, which affects quality of life. Moreover, patients with hematologic disorders may face more severe abnormal uterine bleeding in the laminar air-flow room (LAFR)^11, 12^. In the event of uncontrollable severe vaginal bleeding, the patient's life may be in danger, eventually leading to treatment failure. Currently, the commonly used treatment to stop bleeding in the LAFR is mainly comprises high doses of hormone-based drugs, such as norethindrone, combined oral contraception (COC). However, there is relatively little awareness of the prevention of bleeding in the LAFR.

Gonadotropin-releasing hormone agonist (GnRHa) use in gynecological endocrine therapy can put patients into a pseudomenopausal state. In recent years, the protective effect of GnRHa on the ovary during chemotherapy has attracted attention. Several meta-analyses and prospective randomized studies^13, 14^ have shown that GnRHa significantly reduces the risk of POF in women undergoing gonadotoxic chemotherapy. Although these results seem promising, a paucity of data exists on similar use of GnRHa in the HSCT population, especially in China. Several small studies have suggested an inconclusive benefit of using a GnRHa to preserve ovarian function in the HSCT patients. If GnRHa therapy works for HSCT patients, it will serve as an economical, noninvasive and simple method to protect ovarian function. Therefore, this study analyzed the benefits of GnRHa treatment in premenopausal women undergoing HSCT, including the reduction of vaginal bleeding in the LAFR, protection of ovarian function, and improvement in perimenopausal symptoms.

## Methods

This was a retrospective cohort study. We affirm that: (1) the study was approved by the Ethics Committee of Peking University People's Hospital (No. 2015PHB087-01), including any relevant details; and (2) all experiments were performed in accordance with relevant guidelines and regulations. Moreover, all research was performed in accordance with relevant regulations, and appropriate consent was obtained from all participants and/or their legal guardians and signed by the patients or their families. Patients with hematological diseases at Peking University People's Hospital routinely undergo gynecological physical examination before HSCT, such as the collection of menstrual history, pelvic examination and cervical cancer screening. Patients with a desire for fertility protection are triaged to the gynecological endocrinology clinic for consultation on fertility protection methods. The research subjects of this study were all from the gynecological endocrinology clinic.

Each patient requiring fertility preservation in the gynecological endocrinology clinic was informed by gynecologists about methods of ovarian protection, and was fully informed that the efficacy of GnRHa was not clear. Patients could choose whether to use GnRHa voluntarily, and signed the informed consents. Patients chose to use GnRHa were injected leuprolide acetate 3.75 mg subcutaneously before the start of myeloablative chemotherapy, once every 28 days as a course of treatment. Considering the onset time and safety of GnRHa, patients were advised to get at least two injections of GnRHa before entering LAFR. The gynecological endocrinologists kept the medical records of all patients who planned to undergo HSCT whether GnRHa was used, and reviewed the treatment process of the patients through the medical records.

The clinical data of all patients were collected for analysis when they went returned to the gynecological endocrinology clinic 6–12 months after HSCT. The patients were followed up by assessing vaginal bleeding in the LAFR, menstruation return, menopause-related symptoms, and ovarian function [follicle-stimulating hormone (FSH), luteinizing hormone (LH), and estradiol] were done.

We reviewed data from all HSCT patients for whom medical records were collected in clinic. The study population consisted of women of childbearing age under 40 years of age who were scheduled to undergo myeloablative chemotherapy before HSCT from December 2011 to December 2021. Patients who voluntarily chose to inject GnRHa were included in the treatment group. Patients who chose not to receive GnRHa treatment were included in the control group. The inclusion criteria were as follows: (1) women undergoing myeloablative chemotherapy HSCT for hematological diseases; (2) age < 40 years; and (3) patients with normal hormone levels or without laboratory data but with regular menstruation before HSCT. The exclusion criteria were as follows: (1) patients whose ovarian function was found to have declined before HSCT with myeloablative chemotherapy through menstruation, B-ultrasound monitoring and hormone examination; (2) patients who were unable to survive after HSCT after myeloablative chemotherapy; (3) patients with incomplete clinical data (including patients who were not followed up within 1 year after HSCT); and (4) patients who received less than 2 courses of GnRHa treatment before myeloablative chemotherapy;and (5) patients who were lost to follow-up (more than 1 year after HSCT).

POF was defined as age < 40 years old and FSH ≥ 40 mIU/ml. Patients with ovarian insufficiency underwent hormone-replacement therapy (HRT) after the diagnosis of POF. Recovery of ovarian function was defined as resumption of menstrual cycles (at least two consecutive episodes) without HRT after HSCT and a normal FSH level without HRT. The following conditioning regimens were used and were considered myeloablative: busulfan plus cyclophosphamide; busulfan plus fludarabine; cyclophosphamide plus total-body irradiation (> 10 Gy); carmustine, etoposide, cytarabine, and melphalan; and a single melphalan dose > 140 mg/m^2^.

All data were statistically processed using the SPSS 25.0 (SPSS Inc., Chicago, IL) statistical software package. Continuous variables are herein were described using medians, ranges and ranges; categorical variables are described using frequencies and proportions. Comparisons were performed using the chi-square test, Fisher's exact test, and Mann–Whitney U test. *P* < 0.05 indicated that the difference was statistically significant.

## Results

**Figure 1:**
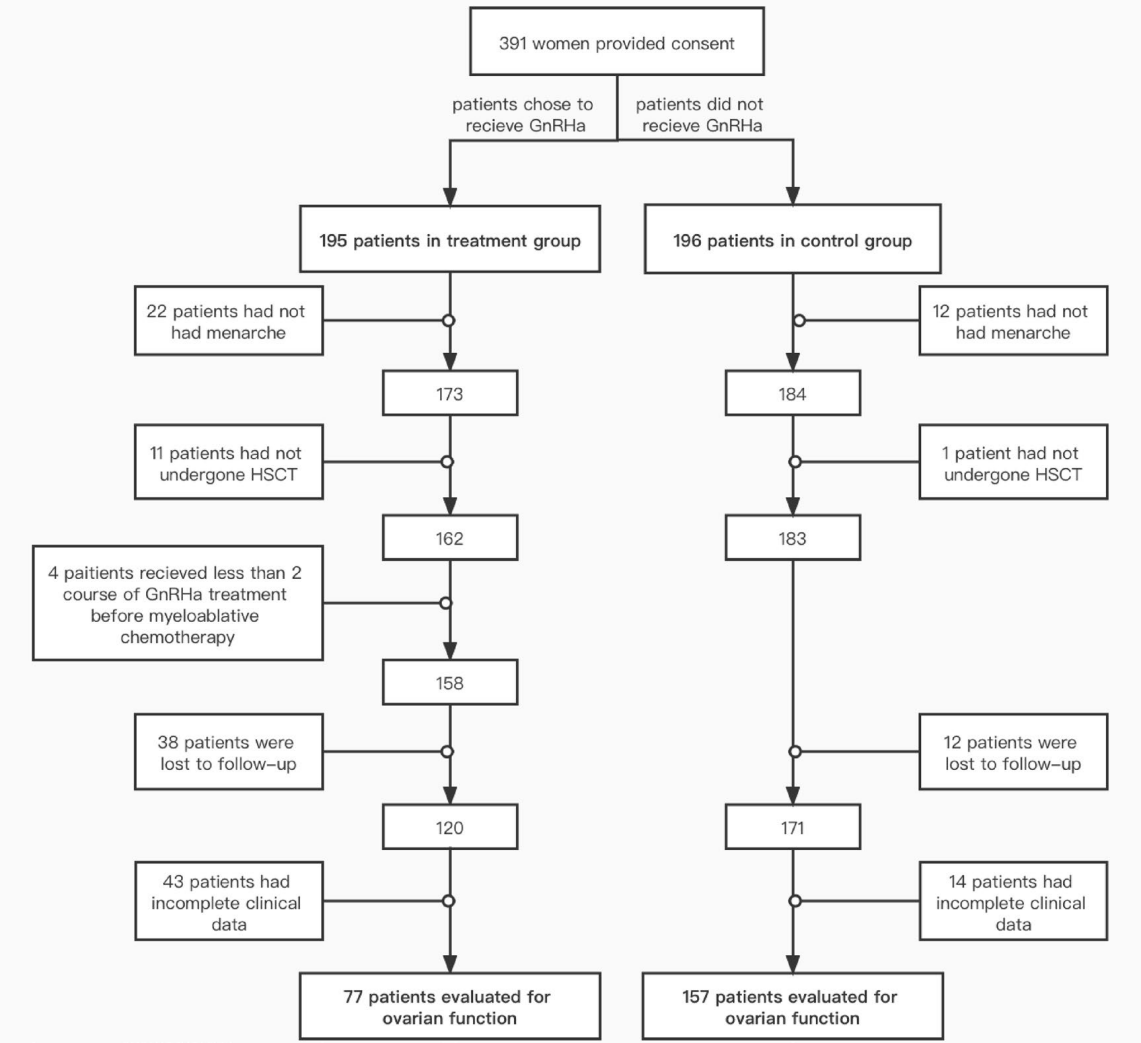
Flow sheet explaining patient dropout. *HSCT* hematopoietic stem cell transplantation, *GnRHa* gonadotropin-releasing hormone agonist.

From December 2011 to December 2021, 391 patients visited Peking University People's Hospital Gynecology Clinic for fertility protection counseling before undergoing myeloablative chemotherapy and HSCT. A total of 234 cases were included in this study, followed up for a median of 28 months. There were 77 cases in the treatment group and 157 cases in the control group (Fig. 1). The general conditions and clinical data of the study population are shown in Table 1. All patients underwent allogeneic transplantation. Except for AA patients who received RIC regimen, the rest of the patients all used MAC regimen. There were significant differences between the two groups in terms of the hematologic disease type and whether they received cyclic chemotherapy before myeloablative chemotherapy and HSCT. Compared with the control group, the treatment group was less likely to receive courses of chemotherapy before myeloablative chemotherapy and HSCT (67.53% vs. 85.35%, *P* = 0.002). There were no statistically significant differences between the two groups in terms of age, HLA- matching status, or graft vs. host disease (GVHD).

**Table 1:** Patient characteristics according to whether GnRHa was injected.

Characterics	Treatment group, n = 77(%)	Control group, n = 157(%)	*P* value
Age(mean ± SD), years	23.83 ± 6.66	24.51 ± 6.79	0.180
Hematologic diseases type			0.017
ALL	25	62	
AML	25	68	
AA	16	13	
MDS	9	8	
Other	2	6	
HLA-matched situation			0.599
HLA-matched	13	31	
HLA-haploidentical	64	126	
Cyclic chemotherapy before HSCT			0.002
Yes	52 (67.53)	134 (85.35)	
No	25	23	
GVHD			0.623
Yes	50	107	
No	27	50	

*P* value indicates the differences between the two groups.

*ALL* acute lymphocytic leukemia, *AML* acute myelogenous leukemia, *AA* aplastic anemia, *MDS* myelodysplastic syndrome, *GVHD* graft versus host disease.

The median follow-up times for the treatment and control groups were 28 and 29 months, respectively. The benefits of patients in the treatment group are mainly to reduce the rate of vaginal bleeding in LAFR and reduce menopausal symptoms. The incidence of vaginal bleeding in the LAFR, in the treatment group was significantly lower than that in the control group (24.68% vs. 79.62%, *P* < 0.001). Compared with those in the control group, the menopausal symptoms of the patients in the treatment group were reduced after transplantation (46.75% vs. 19.75%, *P* < 0.001) (Table 2).

**Table 2:** Observation indexes between the treatment group and control group 6–12 months after HSCT.

	Treatment group, n = 77(%)	Control group, n = 157(%)	*P* value
Vaginal bleeding in LAFR	19 (24.68)	125 (79.62)	< 0.001
Symptoms			
Hot flushes	32 (41.56)	74 (47.13)	0.421
Sweating	24 (31.17)	64 (40.76)	0.155
Nervousness	30 (38.96)	35 (22.29)	0.007
Insomnia	21 (27.27)	74 (47.13)	0.004
Sexual problems	10 (12.99)	40 (25.78)	0.029
None	36 (46.75)	31 (19.75)	< 0.001
Follicles visible on gynecological ultrasound	13 (16.88)	21 (13.38)	0.474
FSH (mIU/ml)	98.00	117.53	0.001
LH (mIU/ml)	65.07	61.38	0.127
FSH < 40 mIU/ml	5 (6.49)	4 (2.55)	0.266
Recovering menstrual Cycles	1 (1.30)	0 (0)	0.329

*P* value indicates the differences between the two groups.

*LAFR* laminar air-flow room, *HSCT* hematopoietic stem cell transplantation, *GnRHa* gonadotropin-releasing hormone agonist, *FSH* follicle-stimulating hormone, *LH* luteinizing hormone.

Hormone levels were measured before HSCT in 35 of 77 patients in the GnRHa group and in 20 of 157 patients in the control group. The data showed no difference between FSH levels and LH levels between the two groups before HSCT (5.69 mIU/ml vs. 5.93 mIU/ml, *P* = 0.582, 5.15 mIU/ml vs. 4.96 mIU/ml, *P* = 0.564). The medians of E_2_ levels were 36.32 pg/ml and 26.38 pg/ml. Observation indices between the treatment group and control Group 6–12 months after HSCT are shown in Table 1. There was no difference in visible follicles by the follow-up ultrasound in the two groups of patients after HSCT (16.88% vs. 13.38%, *P* = 0.474). The level of FSH at 6–12 months after transplantation was lower in the treatment group (98.00 mIU/ml vs. 117.53 mIU/ml, *P* = 0.001), although both levels had reached the POF standard. There was no significant difference in LH levels after transplantation (65.07 mIU/ml vs. 61.38 mIU/ml, *P* = 0.127). Because the sensitivity of the E_2_ assay was 20 pg/ml, most of these levels were not detectable as they were 20 pg/ml or lower. However, the median E_2_ level after HSCT in both groups was less than 20 pg/ml. The proportion of patients with FSH < 40 mIU/ml did not differ between the two groups. Only one patient in the treatment group recovered spontaneous menstruation while none in the control group did (1.30% vs. 0%, *P* = 0.329). That patient was a 25-year-old acute lymphocytic leukemia patient who returned to a regular menstrual cycle with normal hormone levels (Table 2).

Whether or not to perform pretransplant cyclic chemotherapy was associated with the type of hematological disease, and the conclusion remained unchanged after we stratified the main outcome indicators according to the type of disease (Supplementary material).

## Discussion

Our study showed that the use of a GnRHa before transplantation did not preserve ovarian function in patients who underwent HSCT using myeloablative regimens.

Chemotherapy-induced POF was first reported in the late 1950s^15^. Subsequent studies suggested that the effect of ovarian damage may be age dependent and dose dependent^16^. The “Beijing Protocol” using the MAC regimen improved the success rate of allogeneic haplo-SCT, which also meant that high-dose chemotherapy and total-body irradiation were used. The usual MAC regimen for allogeneic HSCT usually includes high-dose alkylating chemotherapy drugs such as cyclophosphamide, and/or total body irradiation (TBI). The commonly used MACs are the classic TBICy and BuCy regimens and their improved regimens. The classic TBICy and BuCy regimens both include cyclophosphamide 120 mg/kg for 2 days and 12–14 Gy TBI for 2 weeks or busulfan 12.8 mg/kg for 4 days. The dose of cyclophosphamide in the improved regimen is 3.6 g/m^2^ for 2 days, and the radiotherapy regimen is a single 770 cGy TBI. Even though the RIC regimen reduces the dose of cyclophosphamide to 90–120 mg/kg for 2–4 days, the dose of chemotherapy drugs used in the regimen is still much higher than that of conventional chemotherapy. Compared with the dose of a course of chemotherapy (for example, the dose of cyclophosphamide in conventional chemotherapy for breast cancer is 0.5 mg/m^2^), the chemotherapy dose in the conditioning regimen will cause more serious damage to the ovary. Treatment-induced POF is a major problem for surviving women of reproductive age. Nakayama et al.^17^ conducted a survey and reported that most patients believed that a discussion of fertility-related or menopausal-related issues was as important as a discussion of their cancer issues and suggested that health care providers should provide information on fertility and menopause repeatedly throughout the treatment period, and that menopause-related information should be reemphasized after HSCT. The American Society of Clinical Oncology guidelines recommend the discussion of ovarian preservation as early as possible in treatment planning^18^.

GnRHa drugs are synthetic peptide drugs modeled on GnRH that are designed to interact with the GnRH receptors and modify the release of gonadotropins. The protective mechanisms of GnRHa drugs on ovarian function remain unclear. Several proposed mechanisms for how GnRHa therapy works have been proposed; these include GnRHa suppression of can suppress gonadotropin levels to stimulate the prepubertal hormonal milieu, which subsequently prevents primordial follicle maturation, reducing the number of follicles susceptible to chemotherapy^19^. Alternatively, a GnRHa can reduce utero-ovarian perfusion, resulting in less exposure of the ovaries to chemotherapeutic agents^20^. Alternatively, it can directly activate GnRH receptors on the ovary and regulate the expression of anti-apoptotic molecules in the gonads^21^. GnRHa therapy has been shown to preserve ovarian function in chemotherapy-treated patients outside of the HSCT setting in meta-analysises and prospective randomized studies^13, 14, 22^. Blumenfeld et al.^23^ presented their meta-analysis of 20 studies (15 retrospective and 5 randomized, controlled trials) that have reported on 1837 patients treated with a GnRHa in parallel with chemotherapy, showing a significant decrease in the POF rate in survivors. In 2015, Moore et al.^24^ published the results of a prospective RCT trial, in which 257 premenopausal breast cancer patients received chemotherapy with or without GnRHa treatment, showing that GnRHa-treated patients had better-preserved ovarian function across multiple endpoints than the controls. However, there are few data on the protection of ovarian function by GnRHa treatment in the HSCT population. Cheng et al.^4^ conducted a prospective phase II study to evaluate the efficacy of a GnRHa in reducing the incidence of POF in the setting of HSCT. Seven of 44 patients (16%) regained ovarian function in the study and they concluded that the use of GnRHa before transplantation did not preserve ovarian function in HSCT patients receiving either myeloablative or nonmyeloablative regimens. However, the study did not include patients who were not injected with GnRHa as controls.

Our study showed that GnRHa treatment applied before myeloablative chemotherapy may be able to reduce the FSH level of patients after HSCT, but it still reaches the standard of POF; this finding revealed that GnRHa cannot reduce the incidence of premature ovarian failure in patients receiving myeloablative chemotherapy HSCT. Only one case maintained spontaneous menstruation, and those hormone levels suggested normal ovarian function, while no such cases were observed in the control group. In conclusion, GnRHa treatment could not reduce the incidence of premature ovarian failure. Combined with previous studies to analyze this study, the following aspects deserve attention. In terms of diagnosis, the diagnostic criteria for POF in this study were age < 40 years old and FSH ≥ 40 mU/ml. However, cyclic HRT was given when the hormone levels reached the POF standard 6–12 months after HSCT, especially in patients with perimenopausal symptoms. In terms of the timing of medication, all patients in this study were given GnRHa before the initiation of MAC. However, considering that some patients with hematological malignancies in the study population received unprotected, gonadotoxic chemotherapy before GnRHa was administered, a stratified analysis was performed. The results showed that the application of a GnRHa did not significantly reduce the incidence of premature ovarian failure, regardless of whether the group was based on disease type or whether there was chemotherapy before MAC.

Although GnRHa treatment does not reduce the incidence of premature ovarian failure, there are other benefits for female patients who use GnRHa before HSCT. The results showed that of GnRHa injections at least 2 courses before transplantation were effective in reducing vaginal bleeding during transplantation. Other studies have also shown that GnRHa treatment may prevent breakthrough bleeding post-transplantation^25^. Once a donor is identified, the recipient completes a pretransplant evaluation and then undergoes a conditioning regimen (1–2 weeks), receives the graft infusion, and then must await the initial signs of engraftment (10–28 days) and repopulation of bone marrow (60–90 days)^26^. Myeloablative conditioning typically results in severe pancytopenia within 1–3 weeks of initiation, and thrombocytopenia increases the risk of heavy menstrual bleeding; potentially life-threatening heavy menstrual bleeding may delay treatment, leading to suboptimal outcomes. Therefore, the menstrual management of patients in the LAFR cannot be ignored. Even without regard to ovarian protection, GnRHa therapy requires only a simple injection and has few side effects when given regularly over 2 courses before pancytopenia, which makes it the first choice of many transplant physicians. Therefore, GnRHa injection before HSCT can be recommended for women of childbearing age to prevent vaginal bleeding during bone marrow transplantation and breakthrough bleeding after transplantation.

Menopause-related symptoms are also important reference indicators for evaluating the ovarian protective function of GnRHa drugs. Previous studies have shown that women with premature ovarian failure who undergo HSCT have lower scores on the symptomatic menopause rating scale and lower modified Kupperman index values than naturally postmenopausal women of the same number of years after menopause^27^. The five most frequently reported perimenopausal symptoms were recorded by the gynecological clinician at the patient's follow-up visit after transplantation. Although GnRHa treatment did not yield a benefit of ovarian protection in terms of diagnostic criteria based on hormone levels, such therapy can significantly reduce the incidence of perimenopausal symptoms; moreover, the role of GnRHa treatment in ovarian protection is difficult to deny, although further research is needed to prove it.

This study has limitations. First of all, it was not a randomized controlled trial (RCT). The patients' exposure history could only be reviewed through medical records, and laboratory examination records were incomplete. This also leads to a high dropout rate of patients due to the loss of follow-up and incomplete clinical data. Second, the final sample size available for analysis in this study was relatively small. Differences in the number of patients with various diseases and differences in the treatment plan and length of treatment before HSCT all increased the possible bias.

## Conclusion

In conclusion, the benefits of GnRHa thrapy can be seen in reducing vaginal bleeding and menopausal symptoms in women of childbearing age who receive myeloablative HSCT. GnRHa drugs may decrease FSH levels after myeloablative chemotherapy, which may suggest that GnRHa treatment has a certain ovarian protective effect but does not reduce the incidence of POF. The clinical application value of GnRHa in fertility-sparing treatment of female patients of reproductive age who are undergoing myeloablative HSCT still needs to be validated by more standardized and rigorously designed RCTs with a larger number of included study populations. However, standardized injection of GnRHa can be considered as a way to prevent vaginal bleeding in the LAFR and improve menopausal symptoms after iatrogenic POF.

### Supplementary Information

Supplementary Tables.

## Data Availability

The datasets used and analysed during the current study available from the corresponding author on reasonable request.
