# Supplementary material for: Analysis of the benefit of gonadotropin-releasing hormone agonist treatment in premenopausal women undergoing hematopoietic cell transplantation
Source: Sci Rep. 2023 Sep 4;13:14497. doi: 10.1038/s41598-023-40778-2 (PMC10477207; doi:10.1038/s41598-023-40778-2)
Supplement: Supplementary file 1 — Supplementary Tables. [file 41598_2023_40778_MOESM1_ESM.docx]

Supplementary Material

## Supplementary Tables

**Supplementary Table 1.** Observation indexes between the treatment group and control group 6-12 months after HSCT in AA patients.

| **AA** | **Treatment Group, n=4** | **Control Group, n=10** | **P value** |
| --- | --- | --- | --- |
| **Vaginal bleeding in LAFR** | 4 | 10 | 0.009 |
| **Symptoms** |  |  |  |
| **Hot flushes** | 8 | 3 | 0.249 |
| **Sweating** | 7 | 9 | 0.433 |
| **Nervousness** | 9 | 0 | 0.001 |
| **Insomnia** | 5 | 5 | 0.714 |
| **Sexual problems** | 2 | 3 | 0.632 |
| **None** | 7 | 7 | 0.715 |
| **Follicles visible on gynecological ultrasound** | 2 | 2 | 1.000 |
| **FSH（mIU/ml）** | 108.91 | 127.95 | 0.273 |
| **LH（mIU/ml）** | 61.45 | 69.00 | 0.511 |
| **FSH＜40 mIU/ml** | 3 | 0 | 0.232 |
| **Recovering menstrual Cycles** | 0 | 0 |  |

P value indicates the differences between the two groups.

LAFR, laminar air-flow room; HSCT, hematopoietic stem cell transplantation; GnRHa, Gonadotropin-Releasing Hormone Agonist; FSH, follicle-stimulating hormone; LH, luteinizing hormone

**Supplementary** **Table 2.** Observation indexes between the treatment group and control group 6-12 months after HSCT in AML patients.

| **AML** | **Treatment Group, n=25** | **Control Group, n=68** | **P value** |
| --- | --- | --- | --- |
| **Vaginal bleeding in LAFR** | 7 | 55 | ＜0.001 |
| **Symptoms** |  |  |  |
| **Hot flushes** | 10 | 30 | 0.722 |
| **Sweating** | 7 | 29 | 0.199 |
| **Nervousness** | 8 | 12 | 0.135 |
| **Insomnia** | 8 | 31 | 0.239 |
| **Sexual problems** | 4 | 19 | 0.237 |
| **None** | 13 | 15 | 0.005 |
| **Follicles visible on gynecological ultrasound** | 5 | 10 | 0.766 |
| **FSH（mIU/ml）** | 103.32 | 105.94 | 0.319 |
| **LH（mIU/ml）** | 64.48 | 53.75 | 0.064 |
| **FSH＜40 mIU/ml** | 0 | 3 | 0.561 |
| **Recovering menstrual Cycles** | 0 | 0 |  |

P value indicates the differences between the two groups.

LAFR, laminar air-flow room; HSCT, hematopoietic stem cell transplantation; GnRHa, Gonadotropin-Releasing Hormone Agonist; FSH, follicle-stimulating hormone; LH, luteinizing hormone

**Supplementary Table 3.** Observation indexes between the treatment group and control group 6-12 months after HSCT in ALL patients.

| **ALL** | **Treatment Group, n=25** | **Control Group, n=62** | **P value** |
| --- | --- | --- | --- |
| **Vaginal bleeding in LAFR** | 5 | 48 | ＜0.001 |
| **Symptoms** |  |  |  |
| **Hot flushes** | 10 | 30 | 0.722 |
| **Sweating** | 7 | 29 | 0.199 |
| **Nervousness** | 8 | 12 | 0.135 |
| **Insomnia** | 8 | 31 | 0.239 |
| **Sexual problems** | 4 | 19 | 0.237 |
| **None** | 13 | 15 | 0.005 |
| **Follicles visible on gynecological ultrasound** | 4 | 8 | 0.972 |
| **FSH（mIU/ml）** | 98.00 | 130.48 | 0.009 |
| **LH（mIU/ml）** | 63.75 | 68.37 | 0.896 |
| **FSH＜40 mIU/ml** | 1 | 1 | 0.495 |
| **Recovering menstrual Cycles** | 1 | 0 | 0.287 |

P value indicates the differences between the two groups.

LAFR, laminar air-flow room; HSCT, hematopoietic stem cell transplantation; GnRHa, Gonadotropin-Releasing Hormone Agonist; FSH, follicle-stimulating hormone; LH, luteinizing hormone

**Supplementary Table 4.** Observation indexes between the treatment group and control group 6-12 months after HSCT in patients.

| **MDS** | **Treatment Group, n=9** | **Control Group, n=8** | **P value** |
| --- | --- | --- | --- |
| **Vaginal bleeding in LAFR** | 2 | 7 | 0.015 |
| **Symptoms** |  |  |  |
| **Hot flushes** | 2 | 5 | 0.153 |
| **Sweating** | 2 | 6 | 0.057 |
| **Nervousness** | 2 | 3 | 0.620 |
| **Insomnia** | 1 | 3 | 0.294 |
| **Sexual problems** | 0 | 3 | 0.082 |
| **None** | 4 | 0 | 0.082 |
| **Follicles visible on gynecological ultrasound** | 2 | 1 | 1.000 |
| **FSH（mIU/ml）** | 95.34 | 103.38 | 0.321 |
| **LH（mIU/ml）** | 76.55 | 47.47 | 0.114 |
| **FSH＜40 mIU/ml** | 0 | 0 |  |
| **Recovering menstrual Cycles** | 0 | 0 |  |

P value indicates the differences between the two groups.

LAFR, laminar air-flow room; HSCT, hematopoietic stem cell transplantation; GnRHa, Gonadotropin-Releasing Hormone Agonist; FSH, follicle-stimulating hormone; LH, luteinizing hormone
